# Pancreatic Cancer Gene Therapy: From Molecular Targets to Delivery Systems

**DOI:** 10.3390/cancers3010368

**Published:** 2011-01-18

**Authors:** Cristina Fillat, Anabel Jose, Xavier Bofill-De Ros, Ana Mato-Berciano, Maria Victoria Maliandi, Luciano Sobrevals

**Affiliations:** Programa Gens i Malaltia, Centre de Regulació Genòmica-CRG, UPF, Parc de Recerca Biomedica de Barcelona-PRBB and Centro de Investigación Biomédica en Red de Enfermedades Raras (CIBERER), Barcelona, Spain; E-Mails: anabel.jose@crg.es (A.J.); xavier.bofill@crg.es (X.B.D.R.); ana.mato@crg.es (A.M.B.); victoria.maliandi@crg.cat (M.V.M.); luciano.sobrevals@crg.es (L.S.)

**Keywords:** viral and non-viral vectors, oncolysis, gene delivery routes, clinical trials

## Abstract

The continuous identification of molecular changes deregulating critical pathways in pancreatic tumor cells provides us with a large number of novel candidates to engineer gene-targeted approaches for pancreatic cancer treatment. Targets—both protein coding and non-coding—are being exploited in gene therapy to influence the deregulated pathways to facilitate cytotoxicity, enhance the immune response or sensitize to current treatments. Delivery vehicles based on viral or non-viral systems as well as cellular vectors with tumor homing characteristics are a critical part of the design of gene therapy strategies. The different behavior of tumoral *versus* non-tumoral cells inspires vector engineering with the generation of tumor selective products that can prevent potential toxic-associated effects. In the current review, a detailed analysis of the different targets, the delivery vectors, the preclinical approaches and a descriptive update on the conducted clinical trials are presented. Moreover, future possibilities in pancreatic cancer treatment by gene therapy strategies are discussed.

## 1. Introduction

**Figure 1.**
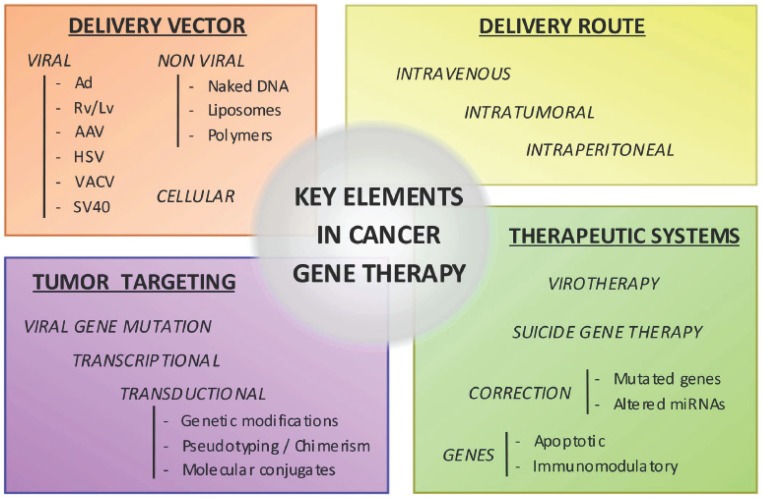
Key factors in the development of gene therapy.

The success of a gene therapy will largely depend on the activity induced by the target genes and the efficiency of gene delivery resulting from the combined effects of the delivery vector and the applied delivery route (Figure 1). Here we present a narrative review that summarizes the recent knowledge on the use of gene therapy for the treatment of pancreatic cancer.

## 2. Target Genes

A large number of genes have been tested for their therapeutic efficacy against pancreatic tumors. The re-introduction of genes capable to activate cell death in tumoral cells or genes that can modulate intrinsic cellular factors and eliminate cancer cells are among the most common approaches. As cancer cells present many up-regulated genes or express mutated genes, silencing or gene-knockdown of undesired genes have also been studied.

### 2.1. Apoptotic Genes

Apoptosis resistance seems to be a hallmark of cancer that largely accounts for the aggressive nature of pancreatic cancer. The re-engagement of the disrupted apoptosis program in pancreatic cancer offers a compelling general strategy for effective and tumor-cell-specific cancer therapy. Studies targeting single apoptosis-associated genes deregulated in pancreatic cancer have shown encouraging results. For example, downregulation of the anti-apoptotic gene BCL-2 triggered anti-proliferative and pro-apoptotic effects in tumor cells but not in non-malignant tissues [1]. Interestingly, inhibition of particular inhibitors of apoptotic proteins (IAPs) such as cIAP-2 and XIAP proved to be very efficient to induce sensitivity to cisplatin, doxorubicin and paclitaxel, common drugs for the treatment of different tumor types, although largely inefficient against pancreatic cancer [2,3]. A novel kind of anti-apoptotic factor in pancreatic cancer has emerged from studies on the circadian clock genes and their influence in tumor progression. In particular, the PERIOD1 (PER1) gene has been recently proposed to be an anti-apoptotic factor in human pancreatic cancer cells. Knock-down of PER1 induced apoptosis and resulted in the upregulation of Bax and downregulation of BCL-2, highlighting its potential interest as a target in pancreatic cancer gene therapy [4].The overexpression of pro-apoptotic genes such as BAX and TRAIL also showed antitumoral effects and sensitization to gemcitabine chemotherapy [5].

### 2.2. Restoration or Inhibition of Gene Mutated Functions

The inhibition of oncogene expression and restoration of tumor suppressor functions have been studied in pancreatic cancer gene therapy. The most common approaches have targeted key mutated genes in pancreatic neoplasia. Strategies involving the silencing of mutated K-ras and/or the functional rescue of p53 or p16 tumor suppressor genes have Shown significant antitumoral responses in mouse models [6-8]. Due to the fact that mutational activation of K-ras is such a common event in pancreatic cancer, targeting of key signaling pathways downstream of mutant K-ras has also been explored through gene therapy approaches. The PI3-kinase/AKT pathway is known to play an important role in maintaining the neoplastic phenotype of pancreatic cancer cells which harbors mutations in K-ras. Interfering with this signaling pathway through the adenoviral gene transfer of a dominant negative inhibitor of mutant Ras reduced tumor growth in nude mice [9].

### 2.3. Suicide Gene Therapy

Suicide or prodrug-converting cancer gene therapy is based on the transfer of an enzyme able to transform a prodrug into a toxic metabolite, resulting in cell death. The herpes simplex virus thymidine kinase (TK) gene in combination with the prodrug ganciclovir (GCV) is the most widely described suicide gene therapy. It consists of the transfer of the TK gene to the tumoral cells, followed by the administration of the nucleoside analogue GCV. TK enzyme converts GCV to its monophosphate form, that is later converted by cellular enzymes into GCV triphosphate and is incorporated into the genome, triggering the formation of double-strand breaks and, finally, causing cell death by apoptosis [10,11]. The cytotoxicity of the system is known to be enhanced by a bystander effect mediated by the transfer of GCV metabolites from the TK-expressing cells to the adjacent cells [12]. TK-mediated suicide gene therapy has been considered for the treatment of pancreatic cancer, as it had been demonstrated to induce relevant cytotoxic efficacy in different models of pancreatic tumors [13,14].

Another active prodrug-activating system is based on the transfer of cytosine deaminase (CD), an enzyme that catalyzes the deamination of cytosine to uracil, and is also able to deaminate the nontoxic 5-fluorocytosine (5-FC) to 5-fluorouracil (5-FU), which is a highly toxic agent. Adenovirus-mediated gene transfer of CD, along with 5-FC treatment, has been shown to inhibit tumor growth *in vivo* [15,16]. Some authors have suggested that CD and TK combined gene expression enhances the ability of the prodrugs to kill cancer cells, with this combination approach being more effective than the treatment of cells with a single prodrug-activating enzyme [17]. However, the activity of both TK/GCV and CD/5-FC systems depend on DNA replication, what could limit their efficacy against slowly growing tumors. Alternatively, the selective activation of purine analogues (6-methylpurine deoxyribose, MePdR) by *E. coli* purine nucleoside phosphorylase (ePNP) has been demonstrated to kill dividing and non-dividing tumor cells [18]. The transfer of ePNP to pancreatic tumor cells renders the cells susceptible to MePdR treatment [19]. Another enzyme used for suicide gene therapy is cytochrome P450, which converts ifosfamide to its cytotoxic form, phosphoramide mustard, and acrolein [20]. Administration of microencapsulated cytochrome P450 2B1 (CYP2B1)-producing cells into tumors and administration of low levels of systemic ifosfamide resulted in tumor reduction in mice models of pancreatic carcinoma [21]. The efficacy of the CYP2B1/CPA antitumoral activity in pancreatic models could be enhanced by the use of CYP2B1 adenoviral vectors retargeted to FGF receptors [22]. Synergistic antitumoral effects have been observed when combined with the TK/GCV suicide approach [23].

### 2.4. Immunomodulatory Genes

Gene transfer into tumor cells has been studied to stimulate immune response against tumor cells. Tumor gene transduction of tumor specific antigens, costimulatory molecules or inflammatory cytokines constitutes the major type of molecules assessed in pancreatic tumors. Vectors expressing IL-1, IL-2, IL-12, TNF-α, GM-CSF have been engineered and have shown significant antitumoral responses [24-27]. IL-12 has also been transferred together with the costimulatory molecule B7.1, and was associated with complete tumor regression in 80% of mice [28]. Combination of restricted replication-competent adenovirus with an adenovirus carrying IL-2 led to a remarkable inflammatory response likely induced by an amplified production of IL-2, and almost complete regression of established tumors [27]. Immune modulation by interferon has also been studied. IFN-γ viral administration provoked an activation of antitumor immunity resulting in complete eradication of both primary and distant tumors [29]. IFN-α and IFN-β also possess direct antitumor and immunomodulatory properties [30,31]. In this line, a combined therapy of recombinant IFN-α with poxvirus vaccines targeting pancreatic adenocarcinomas slowed tumor growth, induced cytotoxic lymphocyte activity, and increased CD8+ tumor-infiltrating lymphocytes [32]. Also noticeable was the induction of tumor regression/stabilization in 50% of treated mice after *in vivo* lentiviral administration of hIFN-β [33].

### 2.5. MicroRNAs

Recent studies have proved that microRNAs (miRNA) are important negative gene regulators controlling a variety of biological processes important in cancer such as proliferation, differentiation and apoptosis [34]. The identification of specific miRNAs signatures in pancreatic cancer revealed aberrant miRNA expression suggesting a role in carcinogenesis [35-37]. Depending on the cancer related genes they regulate, miRNAs could act as tumor suppressors, downregulating oncogenes, or as oncomiRs targeting tumor suppressor genes [38]. The particularity that a unique miRNA may control the translation of a battery of genes participating in common pathways visualizes modulation of microRNA function as a potential therapeutic strategy to specifically kill tumors.

Studies addressing the functional relevance of altered miRNAs and their significance in pancreatic cancer are in early phases. As this field rapidly develops their potential in therapy is also tested. miR-21 has been found to be overexpressed in pancreatic cancers as well as in many other tumor types and it has been associated with a poor clinical outcome [39]. Interestingly, antisense inhibition of miR-21 in cellular models resulted in increased apoptotic response and sensitivity to gemcitabine effects [40,41]. Other up-regulated miRNAs in pancreatic cancer of functional relevance are miR-10 and miR-155. miR-10 has been proven to confer antimetastatic properties in pancreatic and mammary tumor models via suppression of the transcription factors HOXB1 [42]. miR-155 has been proposed as a biomarker for a poor prognosis in pancreatic tumors [43]. It targets tumor protein 53-induced nuclear protein 1 (TP53INP1), resulting in loss of expression and contributing to pancreatic tumor development [44].

Although few studies have been conducted to evaluate the effects of deregulation of miRNAs in pancreatic cancer, the available data already shows its potential in treatment.

## 3. Gene Delivery Vectors

The choice of the vector can largely influence the effectiveness of gene therapy. In this review we summarize the main characteristics of current gene delivery vectors employed to release the genetic material to the target cell. Three major groups can be considered, viral, non-viral and cellular vectors.

### 3.1. Viral Vectors

Viruses have evolved extremely efficient mechanisms to infect cells, subvert cell defenses, deliver their genetic payload, express viral genes, and produce progeny virions. Viral vectors, with either DNA or RNA genomes, have been rendered replication-defective by genetic engineering such that they serve mainly as gene delivery vehicles and do not replicate outside of specialized packaging cell lines. Basically, viruses are transformed to viral vectors capable of delivering genes by substituting key genetic components of the viral genome by the transgene of interest and providing viral genes in *trans* to generate recombinant viral particles.

#### 3.1.1. Adenovirus (Ad)

More than 51 different human adenovirus serotypes have been isolated. Serotype 5, Ad5, has been the most extensively used in gene therapy. It presents low pathogenicity in humans, causing mild acute respiratory infections. Ad vectors have a good safety profile and can be produced at high titers under GMP conditions, do not integrate, can transduce dividing and non-dividing cells and present high *in vivo* transduction efficiency. When administered systemically they are trapped by the liver, which limits tumor delivery and elicits hepatotoxicities. The fact that many individuals have pre-existing neutralizing antibodies against the most common vector strains (Ad2 or 5) can limit gene transfer. Moreover, residual expression of viral genes also causes direct toxicity and the activation of a cellular immune response that leads to the clearance of vector-transduced cells and to short duration of transgene expression [45]. Nevertheless, strategies to overcome such limitations are under development and several preclinical studies employing adenoviral vectors with therapeutic genes, ING4 (inhibitor of growth family) [46], IL-24 [47] or TK [48] have shown different degrees of success in pancreatic cancer models.

#### 3.1.2. Retrovirus/Lentivirus (Rv/Lv)

Originally, retroviral systems were based on the MLV (Moloney Murine Leukemia Virus). They are integrative vectors and offer stable, long-term transgene expression, but only infect dividing cells. In contrast, Lv vectors keep the integrative capacity, but are able to transduce both dividing and non-dividing cells. Rv vectors were initially used to re-establish mutated gene functions such as p53 [49], or to introduce toxic or suicide genes such as nitroreductase gene [50], TK [13,51], p450 [23] or mutated cyclin G1 [52]. In all cases retardation on tumor growth was observed, and in some cases even complete eradication of the tumor. In the more recent years Lv vectors have gained importance over retroviral. Lv vectors transduced pancreatic cancer-derived cells with high efficiency (> 90%) [53]. Lv vectors have been widely used to deliver shRNAs, such as shAPRIL [54], shVEGF [55] or sh-cyclin D1 [56], leading to pancreatic tumor growth inhibition.

#### 3.1.3. Adeno Associated Virus (AAV)

These are non-pathogenic dependovirus thus reliant on adenovirus or herpes simplex virus to complete their life cycle. The various AAV serotypes display different tissue or cell tropism. AAV vectors transduce both dividing and non-dividing cells and show stable expression although they remain episomal. AAV serotypes 1, 2 and 5 transduce pancreatic cancer cell lines *in vitro* poorly when used alone; but co-infection with wild type Ad increased transduction rates dramatically [57]. However, *in vivo* studies have shown good responses. Recombinant rAAV encoding the endostatin gene significantly decreased about 57% of tumor volume in orthotopic pancreatic models [58]. Comparative studies of Ad and AAV vectors carrying the tumor suppression gene fragile histidine triad did not show major differences in tumor suppression by either vector [59].

#### 3.1.4. Herpes Simplex Virus (HSV)

These are enveloped, dsDNA virus with a large exogenous DNA accommodating capacity. HSV-1 vectors are characterized by high efficiency of transduction of several different cell types, both dividing and non-dividing [60]. Moreover, they can be easily produced to high titer and purity without wild type contaminants. Different replication-defective HSV vectors have been produced, but only one preclinical study has been published in pancreatic cancer with a vector expressing a hypoxia-inducible soluble vascular endothelial growth factor receptor (sFlk-) showing an inhibition of angiogenesis and a 59% reduction in the tumoral volume [61].

#### 3.1.5. Vaccinia Virus (VACV)

These are dsDNA viruses able to infect cells of many different origins. Some strains present a natural tropism for tumoral tissue, with a 1,000–10,000 fold higher expression than in other organs. Mutations in essential viral genes have been engineered to improve the safety of the vector when administered systemically [62]. The mechanism of infectivity it is not fully understood, therefore it is hypothesized that the tumoral tropism is based on the entry through tumor cell surface receptors [63]. Systemic administration of a recombinant VACV expressing hIL-1beta in tumor-bearing mice showed a remarkable inhibition of established solid tumors [64].

#### 3.1.6. Recombinant, Simian Virus 40 Vectors (rSV40)

SV40 is a replication defective dsDNA vector that delivers stable transgene expression with high efficiency to many different lineages. An engineered SV40 vector that expresses sst2 somatostatin receptor controlled by human Telomerase tumor-specific promoter was administered intratumoral into Capan-1 tumors and resulted in a marked inhibition of tumor progression [65].

### 3.2. Non Viral Vectors

Non viral vectors are safe, easy to produce at large-scale; they can carry large inserts but they are quite inefficient at transfecting cells *in vivo* [66,67]. Nevertheless several authors have approached their potential to target pancreatic tumors and induce antitumoral effects.

#### 3.2.1. Naked DNA

Naked DNA can be directly injected to the tumor site but it results in poor transfection efficiency, alternatively it can be systemically delivered, although then it is rapidly degraded by serum nucleases [68]. Physical methods that increase the permeability of cell membrane to facilitate the introduction of naked DNA into cells such as electroporation (EP) or ultrasounds have shown to improve its transfection efficiency [69]. Combined suicide TK/GCV therapy with EP in pancreatic tumors showed a signification reduction of tumor growth with 50% complete tumor eradication [14]. Intratumoral injection of the purine nucleoside phosporylase gene followed by fludaravine plus EP led to an inhibition of tumor growth and increased survival [70]. Intramuscular administration of cholecystokinin porcine gene followed by EP into hamsters bearing pancreatic orthotopic tumors led to reduced tumor volume, decreased the number of liver metastasis and enhanced survival [71].

#### 3.2.2. Cationic Liposomes

Cationic liposomes have been engineered to deliver siRNAs or candidate genes to pancreatic tumors. An immunoliposome based complex delivering an anti-HER-2 siRNA has been shown to sensitize tumor cells to chemotherapeutics [72]. A modified liposome with specific ligands for tumor-associated endothelial cells carrying a plasmid encoding thrombospondin-1 eliminated tumors completely after five intravenous injections administered once a week [73]

#### 3.2.3. Synthetic Polymers

Intraperitoneal injections of poliethilenimine (PEI) polyplexes have shown relative efficiency for transgene expression in pancreatic tumors [74]. Upon systemic delivery, aggregation and instability limited cell entrance. Polyplex micelles composed of aligned segments with biocompatible, endosomal escaping, and DNA-condensing functions partially overcame such limitations and gene expression was detected in tumors after intravenous administration [68].

### 3.3. Cellular Vectors

Dendritic cells (DC) are highly effective antigen presenting cells able to stimulate the immune system against tumor-associated antigens [75]. For the treatment of pancreatic cancer, DCs have been engineered to produce interleukins (IL-18, IL-12) or the human tumor antigen mucin (MUC1) [25,75,76]. In all cases, the treatment was well tolerated and an immunologic response was induced, observing partial response in patients treated intratumorally with DC-IL12 [25,76]. Fibroblasts have also been modified to express IL-12 and activate innate immunity [77]. More recently, mesenchymal stem cells (MSC) have gained interest as potential cellular vectors. MSC are pluripotent progenitor cells that are actively recruited to the tumor environment [78-80]. Bone marrow derived-MSC or adipose tissue-MSCs are suitable as gene delivery vectors, easily to expand and can be genetically modified [79-81]. It has been shown that systemic delivery of TK transfected MSC to mice carrying orthotopic syngenic pancreatic tumors significantly reduced the primary tumor growth and the incidence of metastases [80]. Similarly, intraperitoneal administration of MSC-IFN suppressed tumor growth of orthotopic pancreatic tumors [82]. In a combined therapy of MSCsTRAIL with XIAP silencing, remission of subcutaneous pancreatic tumors was achieved [83]. Blood outgrowth endothelial cells (BOECs) are also being tested as gene delivery vectors. BOECs are autologous highly proliferative endothelial cells derived from peripheral blood cultures. When BOECs were modified to express fms-like tyrosine kinase-1 and/or angiostatin-endostatin fusion protein, a reduction by half the volume was observed in established pancreatic tumors [84].

## 4. Oncolysis

In cancer gene therapy there has been growing interest in relying on the efficiency of viral replication itself as a mean to destroy cancer cells in a process referred to as viral oncolysis [85]. The safety and efficacy of this approach depend on selective viral replication in cancer cells rather than in normal cells. Replication-competent viruses can be further modified to express a transgene, originating “armed oncolytic viruses”. Conditional Replicative Adenovirus (CRAds) adenoviruses have already shown antitumoral efficacy in pancreatic xenografts. AduPARE1A, an adenovirus in which the E1A gene was controlled by the urokinase-type plasminogen activator receptor (uPAR), exhibited tumor specificity, reduced toxicity and maintained significant antitumoral activity in pancreatic xenograft models and liver metastasis [86]. ONYX-411 and ONYX-411 armed with K-ras(v12)-specific siRNA, both viruses with tumor selectivity coming from the transcriptional control of the viral genes E1A and E4 by the E2F1 promoter, showed reduced xenograft growth that was more efficient with the armed virus [87]. Arming replication competent adenoviruses with suicide genes, such as TK or the cytosine deaminase enzymes, also provide enhanced antitumoral activity [88,89]. Similarly, CRAds armed with immunostimulatory genes, such as IL-12, achieved an improved antitumor effect [90].

HSV-1 oncolytic viruses have been studied to treat pancreatic cancer. Intraperitoneal delivery of the replication-conditional hrR3 (a mutant containing an insertion of the lac-Z gene in the ribonucleotide reductase gene) followed by GCV, improved survival in a murine model of disseminated pancreatic cancer [91]. In another study, animals treated with the replication competent mutated viruses G207 or NV1020 displayed 25% or 40% complete tumor eradication, respectively [92]. Other promising results were achieved by the mutant HSV-2 virus (FusOn-H2) that replicated selectively in activated Ras signaling pathway, showing how two intraperitoneal injections of the virus at a moderate dose completely eradicated the orthotopic and metastatic tumors in 75% of mice [93]. An armed HSV oncolytic viruses known as Oncovex^GALV/CD^ that combines the expression of the prodrug activating gene [yeast cytosine deaminase/uracil phospho-ribosyltransferase fusion (Fcy∷Fur)] and the fusogenic glycoprotein from gibbon ape leukemia virus (GALV), which aids the spread of the activated prodrug through the tumor, increased tumor shrinkage by five- to 10-fold *in vivo* compared to the non-armed virus [94].

Oncolytic armed vaccinia viruses have also been generated to act against pancreatic cancer. Vaccinia has a natural oncolytic capacity due to the interference of replication in normal cells by the interferon, while the low interferon production in tumoral cells allows virus replication. A replication-competent vaccinia virus (GLV-1h68) that also carries marker genes was systemically administered, and resulted in regression of human pancreatic tumor xenografts [95]. An oncolytic Lister strain of vaccinia virus armed with endostatin-angiostatin fusion gene effectively infected pancreatic tumors and showed significant antitumor potency after intratumoral administration and evidence of angiogenesis inhibition [96].

A variant of measles virus with the ability to enter cells efficiently through the CD46 receptor caused a potent cytopathic effect as a result of syncytia formation. It slowed BxPC-3 xenograft tumor growth and extended survival in mice, although it did not completely eradicate the tumors [97].

Pre-clinical studies have demonstrated that treatment with reovirus, an oncolytic virus selectively able to replicate in cancer cells through exploitation of abnormal Ras signaling is non toxic, inhibits the peritoneal dissemination of pancreatic cancer cells after intraperitoneal administration of the virus and is able to decrease the number of liver metastasis from pancreatic cancer after systemic delivery [98].

The continuous growing of the oncolytic virus field as a therapy in pancreatic cancer treatment provides with *in vitro* studies such as selective tumoral replication of Parvovirus [99], Sindbis virus [100] or Mixoma virus [101] that have been assessed. Moreover, there are a number of studies showing that oncolysis synergizes with particular chemotherapeutic agents and radiotherapy, which highlights its potential as adjuvant therapy [89,102-104].

## 5. Engineering More Effective and Selective Vectors Targeting Pancreatic Cancer Cells

Transduction efficiency and selectivity to the target cells constitute major issues towards the development of efficacious and safe pancreatic cancer gene therapy. Many efforts are being conducted to modify current vectors to efficiently and specifically target pancreatic cancer cells.

### 5.1. Transductional Targeting

This strategy is based on the modification of the vector tropism to produce tumor cell-specific infection. Several approaches have been developed to change the vector tropism.

- *Genetic modification of the proteins participating in viral entry*: In an adenovirus infection the first step in adenoviral entry involves the binding to CAR receptor. Pancreatic tumor cells express limited levels of CAR and this has encouraged research studies to retarget adenovirus entry through cellular receptors abundant in tumoral cells [105,106]. Introduction of the Arg-Gly-Asp (RGD) peptide into the HI loop of the knob domain of the fiber protein targeted adenovirus to alpha-beta integrins and improved transduction in pancreatic carcinoma [107,108]. In a screen of a pancreatic cancer cell lines with a peptide-display adenovirus library, an adenovirus with the SYE ligand was identified as an efficient target vector [109]. Moreover, the YSA peptide that targets the EphrinA2 receptor has also been shown to enhance gene transfer in pancreatic cancer cells [110]. All these improvements in the redirection of viral tropism to pancreatic tumor cells have been achieved *in vitro*, or upon intratumoral injections. However, the disseminated nature of pancreatic cancer would optimally require the application of the gene targeted vectors through intravascular delivery. In this scenario, Ad-based vectors present some limitations due the inherent hepatic tropism, which excludes CAR entry and uses alternate pathways. Studies on the operating mechanisms in the *in vivo* liver adenoviral transduction have shown the relevance of adenovirus binding to blood factors and highlighted the role for Ad5 hexon protein interacting with coagulation factor X as a major mediator of *in vivo* liver transduction [111-114]. Optimized design of retargeting Ad vectors to pancreatic tumors could be engineered by incorporating the genetic modifications for liver detargeting. In retroviral vectors, genetic modifications of the *env* protein have been tested. One example is Rexin-G, a non-replicative-targeted retroviral vector that incorporates a collagen-binding peptide derived from von Willebrand clotting factor, responsible for guiding platelets to injured tissues where collagen is exposed [115]. Transductional genetic retargeting in Sendai virus (SeV) has also been achieved. An optimized oncolytic SeV vector, in which replacement of the trypsin cleavage site of F protein for a urokinase type plasminogen activator (uPA)-sensitive sequence has been generated, showed significant potential to target urokinase-expressing cancers, such as pancreatic cancer. [116].
- *Pseudotyping or* incorporating *chimeric fibers.* Several studies have shown that adenovirus serotypes 11 and 35 present enhanced infectivity compared to Ad5 in pancreatic cancer [117,118]. Moreover, the combination of different fibers in the adenovirus, such are fibers 5 and 35, or fibers 16 and 50 mediated more efficient and specific gene transfer to pancreatic cancer cells [119,120]. In the case of retroviral vectors, pseudotyping with envelope glycoproteins such as the vesicular stomatitis virus glycoprotein (VSV-G) has shown to provide high transduction efficiency for human pancreatic tumors.[51].
- *Molecular conjugates that link the vector with specific cellular receptors.* This approach has been widely explored to modify adenoviral vectors. It is based on the formulation of bispecific conjugates, bearing one component recognizing a region of the adenoviral vector and the other component specific for a cellular receptor, with the adenoviral vector, generating the retargeted vector. Examples are EGFR-retargeted adenovirus, with a bispecific fusion protein composed of a soluble form of truncated sCAR genetically fused to the EGF ligand (sCAR-EGF). The EGFR-retargeted adenoviruses led to enhanced gene transfer efficiency in pancreatic carcinoma cells [108]. Alternatively, a bifunctional reagent has been generated by the fusion of a Fab′ fragment against the adenovirus knob region with the fibroblast growth factor (FGF-2) ligand. This approach resulted in adenoviral vectors retargeted to FGFR positive cells leading to improved transduction of pancreatic tumor cells *in vitro* and *in vivo* [22,121]. Liposomes have also been retargeted by complexing with a single-chain antibody fragment directed against the transferrin receptor and shown to target pancreatic cancer cells efficiently and reached primary and metastatic tumors. [72].

### 5.2. Transcriptional Targeting

Transcriptional targeting utilizes tumor-specific promoters (TSP) for controlling gene expression in the cancer cell. TSP driving the expression of viral essential genes has been tested for the design of oncolytic adenovirus. Promoters from genes altered in a broad number of tumor types and in pancreatic cancer in particular, such as the cyclooxygenase-2 (COX-2), midkine (MK), E2F1, cancer-specific progression elevated gene-3 promoter (PEG-prom), human telomerase reverse transcriptase (hTERT), urokinase-like plasminogen activator receptor (uPAR) have been used to drive E1 and/or E4 adenoviral genes. All of them have shown to be efficient heterologous promoters controlling viral replication in pancreatic cancer tumors.[29,86,122-126].

TSPs have also been used in adenoviral vectors to target cytotoxic transgenes into the tumor cells. The TSP carcinoembryonic antigen promoter (CEA), COX-2, MK or the tissue specific insulin promoter have been used to target TK gene expression in pancreatic cancer [127-129]. The MUC1 promoter has been tested to control human somatostatin receptor subtype2 expression, a pro-apoptotic gene with anti-proliferative effects in MUC1 positive pancreatic cancer cells. [130]. Protein patched homolog 1 (PTCH1), a component of the Hedgehog pathway overexpressed in pancreatic ductal adenocacinoma (PDAC), was used to drive the expression of the antisense SMO, another component of the Hedgehog pathway overexpressed in PDAC [131]. Promoters such as hTERT or PEG-prom have also been tested to specifically target the expression of pro-apoptotic genes Bax and TRAIL, and hPNPase (OLD-35), a gene involved in the RNA degradation and the G2/M cell cycle arrest, to pancreatic tumors [5,132].

In the context of the Herpes Simplex viral therapies, the promoter/enhancer sequence of the tumor-associated antigen DF3/MUC1 has been used to regulate the expression of the gamma 34.5 HSV-1 gene to control viral replication preferentially in pancreatic tumors [133].

### 5.3. Introducing Viral Genes Mutations

This is the principle that guided the initial development of oncolytic viruses. Adenovirus, HSV-1 and HSV-2 have been modified in different genes. Deletion of E1B was carried out to generate ONYX-015, an adenovirus unable to replicate in normal cells, but able to replicate in P53 mutated cells [134]. However, it was later found that ONYX-015 selectivity mostly account from a defect in viral mRNA nuclear export in normal cells that was complemented in tumor cells [135]. ONYX-015 entered clinical trials in pancreatic cancer, but its limited replication capacity has reduced their interest [136]. The viral anti-apoptotic gene E1B19K has been suggested as a potential candidate gene to delete in CRADs. E1B19K is a functional Bcl-2 homolog that binds Bax and Bak, avoiding formation of mitochondrial pores in order to block apoptosis. The ΔE1B19K-deleted mutants synergized with gemcitabine to selectively kill cultured pancreatic cancer cells and xenografts *in vivo* with no effect in normal cells [102]. Since adenoviral replication depends on E1A activity as an inductor of S-phase entry, adenoviruses with partial deletions in E1A (AdDeltaE1ACR2) have been assayed as tumor cell specific lytic agents and shown to be effective in pancreatic tumors [137]. Moreover, a double mutant AdDeltaDelta (DeltaE1ACR2 and DeltaE1B19K) also showed high cell-killing activity in pancreatic carcinomas [104].

A more sophisticated strategy has been designed around the differential response of normal and tumor cells to viral molecules. Adenoviruses present a mechanism to circumvent the blockade in protein synthesis mediated by PKR phosphorylation of the elongation factor eIF2 that takes place in the cell upon viral infection. Adenoviruses synthesize the RNA molecules (termed Viral-Associated RNA, VA RNA) which interact with but do not activate PKR, inhibiting its activation and thus allowing for the synthesis of viral proteins [138]. The rationale for the application of this mechanism as a source of tumor selectivity stems from the observation that oncogenic Ras products can also prevent eIF2 activity and, therefore, complement the lost function in adenoviruses in which the coding sequence for VA RNAs has been deleted. VA deleted adenoviruses have been shown to replicate in cells with activated Ras, as most pancreatic tumor cells, while their replication was inhibited in normal cells by the activation of PKR [139].

Mutations in the Herpes simplex viruses, HSV-1 or HSV-2 gene sequences have also been generated to confer oncolytic activity. For instance, deletion of the HSV-1 gamma 34.5 gene limited replication in tumor cells, although recent studies have shown some degree of neurotoxicity in normal brain cells [140]. Another oncolytic virus, the FusOn-H2 virus, was generated by introducing mutations in the PK domain of the viral ICP10 gene, in this virus replication was dependent on the activation of the Ras signaling pathway [93].

## 6. Delivery Routes

**Figure 2.**
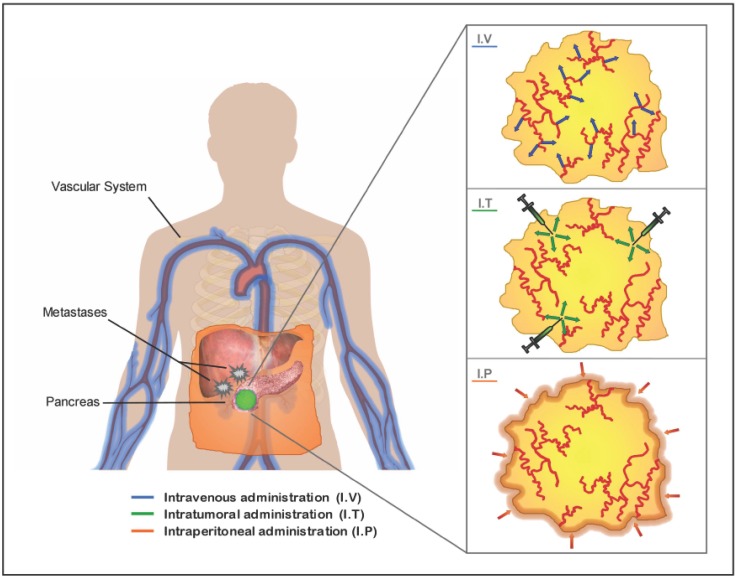
Main delivery routes in gene therapy applications. Representative image of a pancreatic tumor and metastatic foci in the liver. The distribution of the vector into the tumor mass from the main delivery routes is represented in the three right panels. Red lines indicate tumor vasculature. Upon intravenous (I.V.) injection, vectors reach tumor cells through the vascular system. Intratumoral (I.T.) injections can be performed at different sites of the tumor and vector spreads close to the tumor injection site. In intraperitoneal (I.P.) administration, vectors reach tumor cells exposed to the peritoneal cavity.

The location and stage of the tumor will influence the choice of the optimal route of administration to apply. The three major studied routes are: the systemic delivery by intravenous administration specially indicated for pancreatic tumors that present distant metastasis; the intraperitoneal delivery, assessed as a locoregional route to target primary tumors and disseminated peritoneal malignant focus and the intratumoral injection to act against primary tumors (Figure 2).

All routes face with difficulties to achieve optimal delivery. In theory, vascular delivery of vectors will lead to a larger distribution of the therapeutic agent within the tumor. Also, it could provide an option for transducing micro-metastatic tumors. However, vascular delivery, too, has been frustratingly inefficient so far. Often blood vessels are confined to the tumor stroma, and therefore several layers of stromal cells must be passed before malignant cells can be reached by vascularly applied therapeutic agents [141]. Moreover, tumor availability of the vector might be limited by promiscuous association with non target tissues. In the case of adenoviral vectors, 90% of the virus is sequestered into the liver by Kupffer cells and through interaction with clotting factors [142]. Stability and persistence of the vector in the serum and escaping from nuclease degradation is an issue in non-viral vectors. Another hurdle of systemic deliver is the preexistence of a humoral response that could compromise gene transfer [143].

Another commonly studied method of viral vectors delivery to reach pancreatic tumors is intraperitoneal administration. In this case, the vectors do not interact with the components of the vascular system, which reduces some of the hurdles of systemic delivery. However, the physical barriers to reach the bulk of the tumors are increased [125,144].

Intratumoral injections also show limitations to reach the bulk of tumoral cells. The high interstitial fluid pressure in the tumors causes a convective flow that can inhibit the passive diffusion of the vector into the tumor [141,145]. Moreover, the stromal compartment and extracellular matrix components in the tumor act as physical barriers limiting the spread of the vector within the tumor [146]. To overcome these barriers, intratumoral administration of metalloproteases, such as AdMMP8, has shown to reduce the amount of collagen in BxPC3 pancreatic xenografts, facilitating Adwt300 antitumoral activity compared to Adwt300 treatment alone [147]. To improve on vector distribution, multiple injections in different parts of the tumor mass are often applied.

## 7. Pancreatic Cancer Gene Therapy Clinical Trials

A Phase I/II clinical trial of inoperable pancreatic cancer was carried out in a genetically modified-cell based system. Microencapsulated cells carrying the gene CYP2B1 followed by ifosfamide administration were administered to 14 patients, and four regressions and 10 cases of stable disease were reported. Median survival was doubled and one-year survival rate was three-times better than the historic control group [20,21].

A Phase I/II study has been terminated to evaluate the safety of intravenously administered Rexin-G in locally advanced and metastatic pancreatic cancer refractory to standard chemotherapy. Rexin-G is a retroviral vector bearing a cytocidal dominant negative mutant of human cyclin G1. The authors reported no dose-limiting toxicity, no vector DNA integration and absence of replication-competent retrovirus. Also, no vector-neutralizing antibodies were detected. In terms of tumor response, a dose-response effect between overall survival and Rexin-G dosage was observed, with a 28.6% one year survival at the highest dose tested. Thus Rexin-G was safe, well tolerated and contributed to prolong survival in gemcitabine-resistant pancreatic cancer [115]. Importantly, it is currently available as a second-line treatment for a limited number of patients in advanced Phase I/II and Phase II confirmatory trials.

Several trials have focused on the use of vaccines made from gene-modified pancreatic cancer cells to help the body build an immune response to kill tumor cells. In some of the studies, vaccines have been administered in combination with chemotherapy. Two trials have been completed, although the results have not yet been published. One of the studies is a Phase I/II study of an antitumor vaccination using alpha(1,3)galactosyltransferase expressing allogeneic tumor cells engineered by retroviral transduction (clinical trial registration number NCT00255827). Its aim was to establish the proper vaccine dose, examine side effects and potential benefits of the treatment evaluating tumor and immunological responses. The expression of the alpha(1,3)galactosyltransferase enzyme will result in the incorporation of alpha-gal epitopes on membrane glycoproteins and glycolipids increasing their immunogenicity by triggering a hyperacute rejection response. The other trial is a Phase II study evaluating the safety and efficacy of allogeneic pancreatic tumor cells genetically modified to express the GM-CSF factor with chemoradiotherapy for resected stage I or stage II adenocarcinoma of the pancreas (NCT00084383). Two additional clinical trials based on the same vaccine but with different combined therapies are also ongoing.

Early Phase I/II clinical trials reported the feasibility to apply intratumoral injections of an adenoviral vector carrying the human tumor necrosis factor (TNF)-alpha gene regulated under the control of a radiation-inducible gene promoter TNFerade™ (Genvec, Inc.) followed by chemoradiation. A recently published case report on a patient demonstrated the capacity of the therapy to shrink the tumor, facilitating later operability to resect the tumor [148]. However, a Phase III clinical trial (NCT00051467) of TNFerade has been recently discontinued with the argument that an interim analysis of overall survival indicated that the trial will not meet the goal of demonstrating persuasive evidence of clinical effectiveness. On the other hand, a Phase II clinical trial aiming to demonstrate the benefit of TNFerade plus radiation followed by autologous dendritic cells *versus* radiation only followed by cell vaccine injection is still ongoing (NCT00868114).

A Phase I study based on the use of a recombinant adenoviral vector expressing the TK gene (AdTK) followed by valacyclovir is recruiting patients. There are two arms of treatment. Arm A is for resectable tumors. AdTK will be given before intratumoral surgery by computed tomography (CT) or endoscopic ultrasound (EUS)-guided plus 14 days of valacyclovir pills. A second AdTK dose will be given into the tumor bed at the time of surgery followed by 14 days of valacyclovir. Arm B is for locally advanced tumors. AdTK will be delivered by CT or EUS-guided injection into the tumor previous to chemoradiation and at week three of chemoradiation. Both injections will be followed by 14 days Valacyclovir (NCT00638612).

The use of replication-competent viruses is also under clinical trials. The first study was a Phase I/II carried out with repeated intratumoral administrations of ONYX-15 adenovirus by EUS-guided. The trial showed the feasibility and tolerability of the therapy. However its very limited replication capacity compromised its antitumoral effect [136].

A Phase I study combining suicide gene therapy delivered by the replication competent adenovirus expressing the TK and the CD enzymes (Ad5-yCD/mutTKSR39rep-ADP) with chemoradiation therapy is currently recruiting patients with non-metastatic pancreatic adenocarcinoma. A single intratumoral injection EUS-guided followed by three weeks of 5-FC and valacyclovir plus six weeks of capecitabine and 54 Gy radiation will be applied to patients with non metastatic non-resectable tumors. Five dose levels of adenovirus will be tested and the primary endpoint will be toxicity (NCT00415454).

A Phase I study is ongoing to assess the safety and effectiveness of intratumoral injections of the oncolytic Herpes simplex virus, OncoVEX GM-CSF into unoperable patients with pancreatic cancer (NCT00402025).

Regarding non-viral delivery cancer gene therapy, a Phase I dose escalation study to assess the safety and tolerability of a cholesterol liposome-based nanoparticle carrying the pro-apoptotic *Bik* gene to patients with advanced pancreatic cancer is being placed (NCT00968604).

## 8. Future Perspectives

A broad number of pancreatic cancer gene therapy strategies have been studied and are at different levels of development. For a large group of them promising preclinical data has been generated although a limited number of clinical trials have been carried out, which highlights the difficulties to move from the bench to the clinic. The nature of pancreatic cancer, with a high percentage of cases presenting some level of dissemination at the time of diagnosis, brings the necessity to deliver therapies through loco-regional or systemic routes. In line with this, the development of selective vectors is of great interest, and as presented in the review, is an active area of research, particularly related to oncolytic viruses that are among the most promising candidates to further develop in clinical application. To continue with the generation of more potent and selective antitumoral gene therapies for pancreatic cancer that can have a strong impact in the clinic, the incorporation of the basic knowledge that tumor biology is constantly providing will become fundamental.

Recent theories suggest that a small population of cells within some tumors possess the ability to self-renew and proliferate and are thus able to maintain the tumor mass. These cells, which are called tumor-initiating cells or cancer stem cells (CSCs), have been observed to share certain characteristics with normal stem cells, specifically the ability to give rise to all cell types found in a particular cancer sample [149,150]. CSCs are therefore tumorigenic, are proposed to persist in tumors as a distinct population and cause relapse and metastasis by giving rise to new tumors [151]. Recent evidence supports the existence of human pancreatic cancer stem cells (PCSC), which appear to drive tumor initiation and progression and are particularly resistant to cell death induced by radiation or chemotherapy [152,153]. The identification of pancreatic cancer stem cells and further elucidation of the signaling pathways that regulate their growth and survival may provide novel gene-therapy based therapeutic approaches to treat pancreatic cancer.

Studies to define the signaling networks in PCSCs will be needed to determine which targets are most likely to be effective. One approach to target stem cells is certainly the inhibition of stem cell-associated pathways (e.g. sonic hedgehog, mTOR, notch, BMI, BMP). Pharmacological studies are beginning to highlight the potential to target such molecules. For the first time, a multimodal therapy, involving the inhibition of two relevant stem cell pathways then SHH and mTOR pathways and additional chemotherapy resulted in a significant depletion of the PCSC pool reducing tumorigenic and metastatic activity, and long-term event-free survival [154].

Because CSCs possess many of the features of normal stem cells, it will be important to determine if such targeting strategies may be effective in tackling CSCs and discriminate from normal stem cells. This would, of course, be a prerequisite for the clinical application of new treatment modalities. We could envision that gene therapy with specific modifications on the vectors, based on CSC characteristics, can further provide a selective control to preserve normal stem cells.

Gene therapy may then become a potent weapon contributing to pancreatic cancer treatment, although not necessarily as a unimodal treatment, but in synergy with already existing cancer therapies.
